# Telaprevir or boceprevir for hepatitis C treatment: a first survey on pharmacoutilization

**DOI:** 10.3389/fphar.2013.00114

**Published:** 2013-09-17

**Authors:** Maurizio Capuozzo, Alessandro Ottaiano, Eduardo Nava, Valerio Scotti, Stefania Cascone, Adriano Vercellone, Claudia Cinque, Rosario V. Iaffaioli

**Affiliations:** ^1^Department of Pharmacy at the Local Sanitary Agency NA 3 SouthNaples, Italy; ^2^Department of Colorectal Oncology at the National Cancer Institute, “G. Pascale” FoundationNaples, Italy; ^3^Malzoni Radiosurgery CenterAgropoli, Italy; ^4^Pharmacist at the Local Sanitary Agency NA 1 CenterNaples, Italy

**Keywords:** HCV, boceprevir, telaprevir, pharmacoutilization, survey

The treatment of hepatitis C has dramatically improved over the last decade. The association between pegylated interferon and ribavirin is the first-line gold standard but it has some limitations: (i) a limited efficacy in patients with hepatitis C virus (HCV) genotype 1 and (ii) an unfavorable side effect profile. Development of new therapeutic approaches is warranted (Manns et al., 2006). Generally, the treatment of chronic HCV infection is focuses (i) to achieve sustained eradication of HCV [sustained virologic response (SVR): persistent absence of HCV RNA in serum 6 months or more after completing antiviral treatment] and (ii) to prevent progression to cirrhosis and hepatocellular carcinoma (HCC). Currently, the most promising drugs against HCV infection (genotype 1) are protease inhibitors. They are peptidomimetic inhibitors of the HCV non-structural (NS) 3/4A serine protease. NS3 protease plays an important role in the HCV life-cycle by causing cleavage of HCV polyprotein at the NS3-NS4A and other downstream junctions (Tomei et al., 1993; Romano et al., 2012).

Telaprevir and boceprevir were approved by the Food and Drug Administration (FDA) in May 2011 for the treatment of HCV genotype 1 in combination with peginterferon and ribavirin (triple therapy) in adult patients with compensated liver disease, including cirrhosis, who have not been treated before or who have failed a previous treatment (Asselah, 2012; Popescu et al., 2012).

In Italy, telaprevir and boceprevir were approved in December 2012 after a complicated prescriptive pathway (definition of the AIFA—Agenzia Italiana del FArmaco—register for the intensive monitoring, identification of authorized centers for prescription, definition of dispensing modalities).

The first prescriptions of telaprevir and boceprevir in the Local Sanitary Agency (LSA) Naples 3 South Italy (i.e., LSA, NA 3 South, 1.200.000 inhabitants, Campania Region) were done in March 2013. Currently (June 2013), patients treated with the protease inhibitors are 87: 58 with telaprevir (51 naive and 7 null responders) and 29 with boceprevir (24 naive and 5 null responders). During the observed 4 months, 8 treatment interruptions have occurred, all with telaprevir. Reasons for interruption were: 2 cases of severe anemia, 1 case of severe allergy with rush. Five patients were lost at follow-up. No interruption occurred among patients receiving boceprevir. This first survey of pharmacoutilization clearly shows that telaprevir is more frequently prescribed than boceprevir.

Probably, this is due to different therapy protocols. In fact telaprevir is indicated in triple therapy for the first 12 weeks followed by a dual therapy (only with peginterferon and ribavirin) for 36 weeks.

Boceprevir is started after 4 weeks of a dual therapy with peginterferon alfa and ribavirin. The combination therapy (boceprevir, peginterferon and ribavirin) is administered for 24 weeks if the virus is undetectable at week 8 and 24 or for 44 weeks if the virus is detectable at week 8 but undetectable at week 24.

Both drugs achieve similar SVR rates but treatment strategies are completely different. The therapy with telaprevir appears easier and faster. In this first 4 months, the total pharmaceutical spending to acquire protease inhibitors for 87 patients was approximately € 1.700.000. In particular, € 1.350.000 were spent for telaprevir and € 350.000 for boceprevir.

The choice on a specific protease inhibitor should weigh several factors including the treatment strategy, the duration of therapy, the likelihood of achieving a SVR, the safety profile and the costs (Esteban and Buti, 2012). We are really concerned about the high cost of the therapy with protease inhibitors. In our series we observed that among patients who interrupted the treatment 1 was male and 7 female; this could suggest that gender could be associated with treatment compliance. However, we cannot rule out any conclusion and studies on large series are warranted to find predictive factors for response to protease inhibitors in HCV.
